# Monitoring of Civil Infrastructures by Interferometric Radar: A Review

**DOI:** 10.1155/2013/786961

**Published:** 2013-09-09

**Authors:** Massimiliano Pieraccini

**Affiliations:** Department of Information Engineering, University of Florence, Via Santa Marta, 3, 50139 Firenze, Italy

## Abstract

Ground-based radar interferometry is an increasingly popular technique for monitoring civil infrastructures. Many research groups, professionals, and companies have tested it in different operative scenarios, so it is time for a first systematic survey of the case studies reported in the literature. This review is addressed especially to the engineers and scientists interested to consider the applicability of the technique to their practice, so it is focused on the issues of the practical cases rather than on theory and principles, which are now well consolidated.

## 1. Introduction

Radar interferometry is a powerful remote sensing technique, able to detect small displacement at great distance. The scientific background is derived from space technology. Since 1990s [1], satellite-based radar has been able to exploit the phase information of images for detecting ground displacements of a few millimetres at a distance of hundreds of km. These developments had an early followup in analogue ground-based radar systems in the late ‘90s. In 2000 Tarchi et al. [2] proposed for the first time exploiting the ground-based interferometry principles for monitoring buildings. In 2004, Pieraccini et al. [3] designed and tested in field the first interferometric radar for dynamic monitoring of bridges. Since those early days, the technique and the equipment have been continuously developed and tested in different operative scenarios until their diffusion in the community of structural engineers and scientists.

## 2. Working Principle of Radar Interferometry

**Figure 1:**
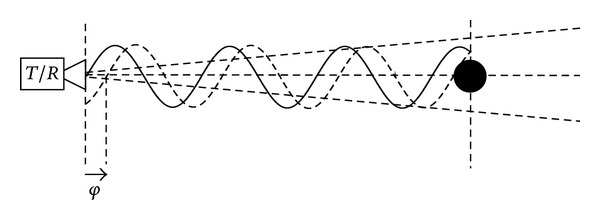
Working principle of interferometry *T*/*R*: transmit/receive equipment, *φ*: phase.

An interferometric radar is a sensor able to detect differential displacements of the targets in its cone of view by exploiting the phase information of the backreflected microwave signal. In Figure 1 its working principle is sketched. 

If we consider a monochromatic wave impinging a single point scatter, the backreflected wave has a phase difference (with respect to the transmitted one) depending on the distance between radar and target. This phase information cannot be exploited directly for distance measurement, as it is affected by an ambiguity equal to half a wavelength (i.e., distances that differ by a multiple of a half a wavelength give the same detected phase), but if the target moves of a fraction of wavelength, the differential displacement can be detected as a phase shift with a precision depending on the capability of the electronic device to appreciate small phase rotation. In practice, by using 17.6 mm as wavelength (corresponding to the central frequency of the microwave band licensed for this kind of applications), with an integration time of 10 *μ*s, the displacement precision is in the order of 0.1 mm. Better precision (up to tens of *μ*m) can be obtained with longer integration time, which can be set when the motion to be detected can be considered stationary (this is the norm when the purpose is to detect the natural frequency of the stationary modes of a structure).

**Figure 2:**
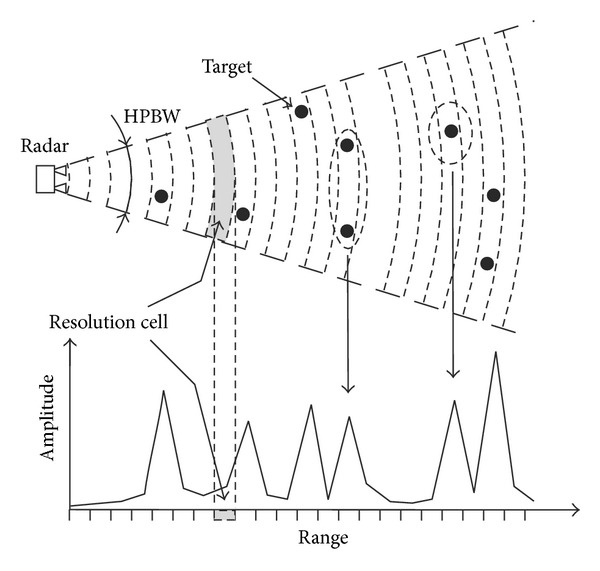
Interferometric radar. HPBW: half-power beamwidth.

The same working principle is applicable when the radar transmits a modulated signal that gives to the sensor the capability to detect separately the displacements of different targets (or portions of targets) at different distances from the radar head, which provided that they are spaced more than the resolution of the radar (Figure 2).

Only-range resolution could be not enough in any application. In order to provide angular resolution too, it is necessary for rotating or moving the radar head. In this way it is possible to obtain a bidimensional image in which the responses of targets at the same distance from the radar can be discriminated in azimuth [2, 4]. In other words, moving the radar head, it is possible to obtain a bidimensional image of the displacement pattern and not only a plot of its projection in the view direction. This is an important capability, but obviously it makes the sensor rather slow, heavier, and bulky, so it is an option not so commonly implemented.

Generally speaking, an interferometric radar can be used both for dynamic and static tests of engineering structures, but aims and scopes of the two modalities are quite different. In the first case the engineer is interested mainly in the frequency spectrum of the displacement. In the second case, the structure is in static condition, and the aim of the measurement is to detect precisely the displacement in relationship to a dead load. But, while frequency spectrum measurement does not present critical issues (at least in certain conditions, as we shall see), the measurement of the absolute displacement by radar interferometry is affected by a systematic and deterministic error that can be kept small but cannot ever be entirely eliminated. Indeed, the target is never a single electromagnetic scatter (an ideal isotropic reflector) but a complex structure. Its movement changes slightly the geometric shape altering its interaction with the impinging electromagnetic wave. This does not affect the measurement of frequency, but it could affect the absolute measurement of displacement with an error that can range from a few percent to even 10–20% depending on particular shape and movement of the structure under test. As rule of thumb, the larger the displacement, the greater the percentage error. Another essential operative difference between dynamic and static tests is that in the second case the measurement time is long enough to allow to rotate or move the radar head, so it is possible to obtain a bidimensional image of the displacement pattern. 

## 3. Dynamic Monitoring 

As mentioned above, a radar used for dynamic monitoring of an infrastructure cannot provide a bidimensional map of the displacement but only a plot in view direction. Therefore, the engineering structures more suitable for this kind of sensor are those with a prevalent dimension, such as bridges (that span mainly in horizontal direction) or towers (that span mainly in vertical direction). 

### 3.1. Bridges

**Figure 3:**
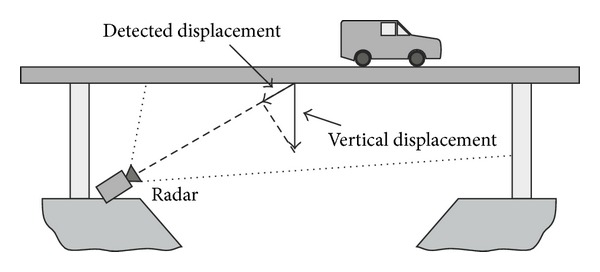
Interferometric radar for monitoring a bridge.

The standard arrangement for monitoring a bridge using an interferometric radar is sketched in Figure 3 [3]. 

**Figure 4:**
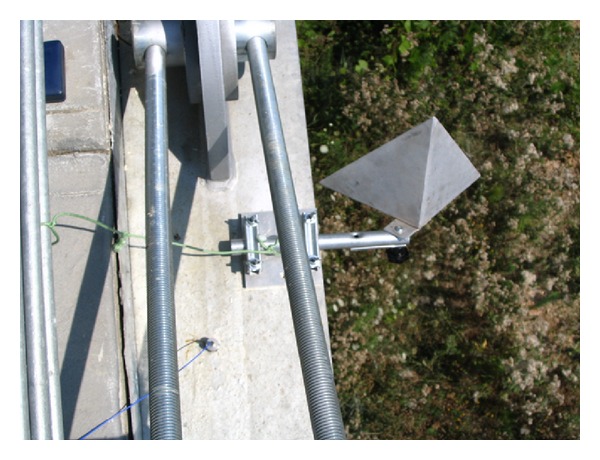
Corner reflector installed on a bridge.

The instrument is positioned at the base of a pier in order to image the lower part of the deck. The antenna's lobe (HPBW: half-power beamwidth) is large enough to illuminate a significant part of the arcade, so different sections of the bridge arcade can be discriminated by their different distances from the sensor. The radar gives a plot of the backreflected amplitude (as in Figure 1), which shows a series of peaks. Each of these corresponds to a geometrical feature of the arcade able to reflect electromagnetic waves, and it can be a transversal beam, a corner, or even just a bolt. In correspondence of each peak the sensor is able to detect the displacement component along the view direction. The deformation curve can be obtained by interpolation [6]. As mentioned above, the correspondence between peaks and points on the bridge relies on the measured distance from the radar, but in particular application a more precise location of the measurement point can be necessary. In these cases, a special reflectors should be installed on the bridge arcade. These are metallic trihedrons (named “corner reflectors”) with a side typically of 20–50 cm (see Figure 4).

Their particular shape has the property to reflect an impinging wave at the same direction from which it had come. As their reflectivity is very high, they are clearly identifiable in the radar plot. The evident drawback in the use of corner reflectors is that they have to be installed on the structure, while a key advantage of the radar is just the capability to operate remotely. 

In order to assess the health of an infrastructure, the detected movement of a bridge has to be correlated to its vibration modes, and it can be done using the standard mathematical procedures developed for the accelerometers [7].

**Figure 5:**
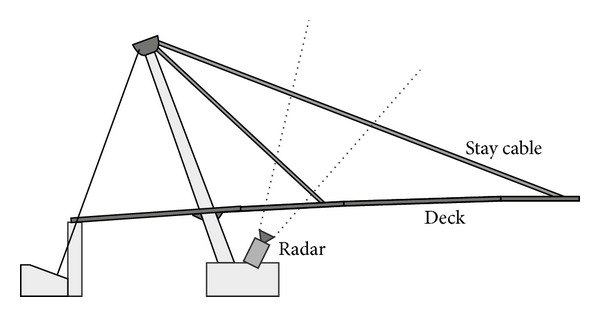
Monitoring of stay cable with interferometric radar.

In cable-stayed bridges the radar can be used for monitoring even the stay cables. Indeed, the tensional state of the cables affects their oscillation frequency, which can be detected by the radar without installing any device on the cable [8], as shown in Figure 5 where the radar is pointed to the cables from a ground installation.

It is important to note that the possible torsional movements of the deck are not detectable by radar in standard configuration. This can be a severe drawback of the technique. Nevertheless, Dei et al. [9] proposed a method for detecting even possible torsional modes. The basic idea is to use a radar able to acquire a radar image of the deck by moving it along a mechanical guide [4] or by rotating the head [32]. This radar image is used as reference for fitting the measured dynamic data with a model that takes into account even possible torsional movements. The main drawback of this procedure is the need of a radar with imaging capability (that is much more heavy and expensive then a radar head mounted on a tripod) and even the complexity of the two-phase measurement. Furthermore, the robustness of the inversion algorithm should be proven in a larger number of cases. 

### 3.2. Masonry Towers

**Figure 6:**
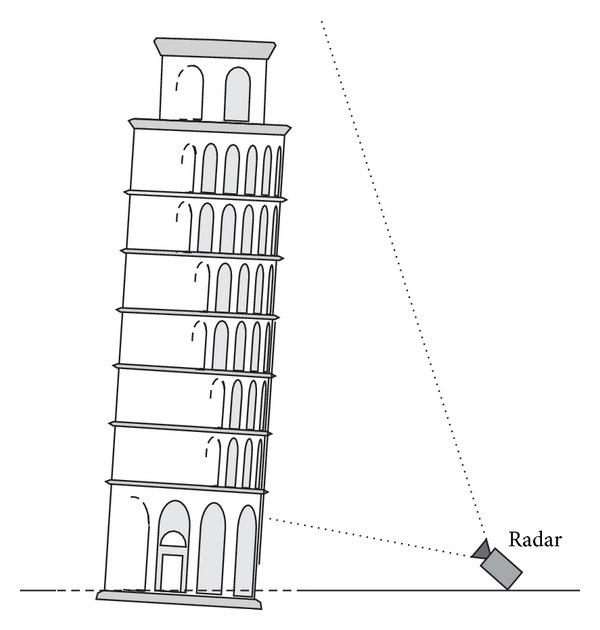
Radar measurement of Leaning Tower of Pisa.

The towers are structures rather vulnerable that need constant surveillance. Ancient bell towers, minarets, and lighthouses, are often part of World Heritage and so their conservation is a priority. Aging of materials, long-term ground subsiding, and accumulation of the effects of earthquakes and strong winds, as well as vibrations induced by today's vehicular traffic, are all causes of possible deterioration. Dynamic characterization is recognized as a powerful method for testing the conservation status of structures, and in planning maintenance or repairs [33]. Indeed, as the resonance frequency of a structure is directly related to its rigidity, its measured value can highlight a damage that may compromise the structure's integrity [34, 35]. In effect, the interferometric radar has been successfully tested in famous historic monuments as the Giotto's tower [13] in Florence (Italy), Mangia's tower [14] in Siena (Italy), and the Leaning Tower of Pisa [15] (Italy). As an example, Figure 6 shows the measurement geometry in the radar monitoring of the Pisa Tower.

Due to the asymmetric shape of the tower produced by its inclination, four different positions of observation were chosen. The radar was oriented empirically at an elevation angle such that the antenna half-power beamwidth illuminated most of the tower surface. The vibration frequencies were evaluated separately from each radar position. Each single measurement spanned 1800 s. It has been revealed a resonance frequency of 1.04 Hz along the west-to-east direction, slightly different from the frequency of 1.01 Hz along the north-to-south direction. These values coincide substantially with the frequencies 1.06 Hz (W-E) and 0.98 Hz (N-S) measured by other authors using microtremor measurements [36]. The case of Pisa's Tower is emblematic, but the presence of two resonances (i.e., two first modal frequencies) is rather common. A perfectly symmetric base (a circle, a square, an hexagon) is rare, as a small difference between the side lengths, or even an external constrain as a building leaning against, can break the symmetry. 

### 3.3. Wind Turbines

**Figure 7:**
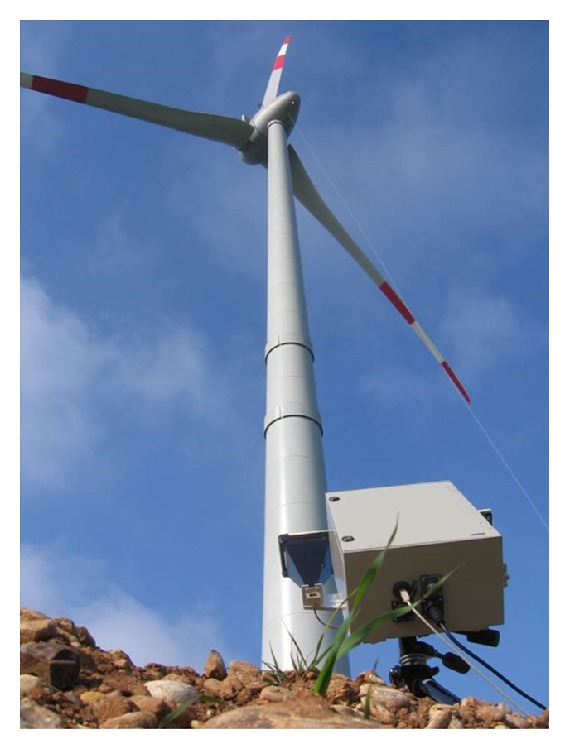
Radar measurement of a wind tower.

The number of wind turbine towers installed worldwide is over several hundreds of thousands and is fast growing [37]. All these structures have to be tested after installation, and they need a periodical monitoring. The key requirements for this kind of in-field testing are (1) in-service operation, (2) equipment portable and fast, and (3) direct measurement of the deflection of the structure under test. These requirements are fulfilled by the interferometric radar, which has been extensively used in this application [17–19]. The movement of the blade is not a problem if the measurement is limited to the points of the tower lower than the blade edges or if the blades are stopped. As an example, Figure 7 shows an interferometric testing of a wind tower. The joints between the sections (one of 3-4 meters) are good point scatters for the electromagnetic wave impinging the structure. Each of these gives a well-detectable signal so it acts as a sort of “virtual sensor” installed on the tower.

**Figure 8:**
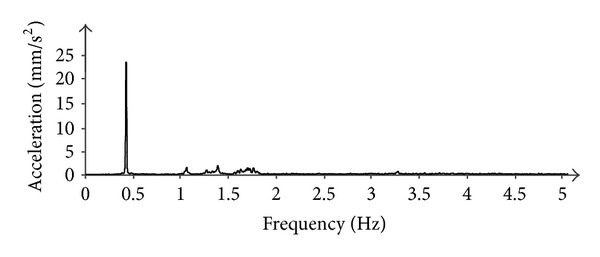
Spectral power distribution of the movement of a wind tower.

Figure 8 shows the spectral power distribution of the acceleration measured by radar in a point at 72 m height of a wind tower. The speed of wind was about 11 m/s as measured by the anemometer of the wind turbine. The radar was installed at 14 m from the base of the tower. 

**Figure 9:**
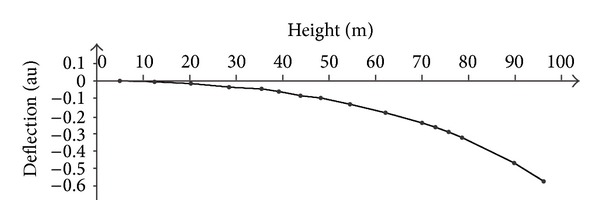
Modal shape at 0.43 Hz.

**Figure 10:**
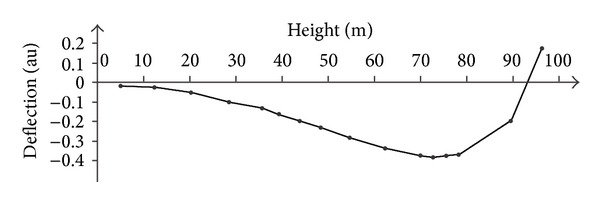
Modal shape at 1.06 Hz.

As the radar is able to provide simultaneously the movement of several points on the tower, it is possible to estimate the modal shape of the main modes as shown in Figures 9 and 10. 

In a wind field there could be even 50–100 towers, therefore Pieraccini et al. [17] proposed an operative modality (named “landscape”) for measuring at the same time several towers, and provided that they can be seen from a single point. Nevertheless in this modality the movement of the blades give additional frequencies (due to the rotation speed) which can be confused with the frequencies of the structural modes. 

### 3.4. Chimneys

The chimneys of industrial plants are high and thin structures sensible to the wind, that can give severe problems when it triggers the detachment of von Kármán vortices. Several authors [10, 20–22] reported the use of the interferometric radar for detecting the oscillation of chimneys. Although these structures are often already instrumented with an accelerometer, the radar can provide the additional capability to detect the whole modal shape sampled in many points.

### 3.5. Antenna Masts, Lighting Towers, and Streetlight

Antenna masts, lighting towers, pylons, and streetlights are all steel structures that are potentially dangerous if they are not periodically monitored. Currently they are occasionally inspected, but there are no satisfactory monitoring tools for expeditious and affordable operations. In this context, the interferomeric radar can offer a fast no-contact measurement device already successfully used for this kind of applications [11, 23].

### 3.6. Culverts

**Figure 11:**
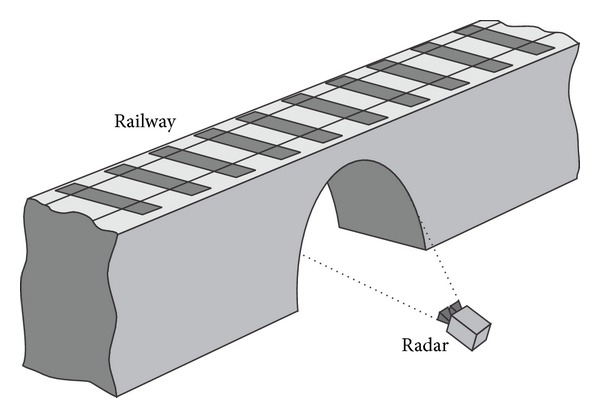
Railway culvert monitored by interferometric radar.

The railway culverts are routinely tested to validate structural specifications or to provide diagnostic surveys for planning maintenance. Beben [24] used the interferometric radar to study deeply the dynamic impacts of the service loads (kinematic excitations during passage of the real trains) on the corrugated steel plate culverts. A sketch of the experimental setup is shown in Figure 11. 

Measurements were made for all trains passing over the culvert during a 24 h period. Finally a comparison between the dynamic amplification factor (DAF) obtained from tests and the dynamic coefficients, as reported in three design bridge standards, has been presented.

### 3.7. Buildings

A microwave interferometer has been employed to remotely detect the oscillations induced by vehicular traffic on the dome of the Baptistery of S. Giovanni in Firenze (Italy) [30]. The measurement has been carried out to assess the reduction of the structure oscillations after the ordinance issued by the Major of Florence forbidding all kind of vehicular traffic in the square around the Baptistery. The estimation of the structural vibrations was performed by using the recommendations of Italian UNI9916, and it resulted that “peak component particle velocity” had a decrease of about 33% from before to after the traffic block.

Recently (2013), Negulescu et al. [31] used the radar interferometry in a large architectonic complex. The building includes a double basement, a ground floor, and 10 storeys. It has a curved form comprised of a central building with two asymmetric wings with different lengths and inclinations. In such an articulated structure, the identification of the vibration modes required particular care in data analysis. 

## 4. Static Monitoring 

The static test is a routine procedure for bridges and floors. Its scope is to detect the displacement in relationship to a dead load. These kinds of measurements are critical issues for the dams that are equipped with numerous and even redundant instrumentation. As it is not necessary for sampling time to be short enough to detect vibration and transient events, the interferometric radar can used in both modalities: only-range (i.e., able to provide only a plot in view direction) and cross-range (i.e., able to provide a bidimentional map of the displacement).

### 4.1. Bridges

Normally the radar is positioned at the base of a pillar in order to image the lower part of the deck as shown in Figure 3. Statistic tests require to load and unload the deck (using trucks for road bridge, train for railway bridge, or bins full of water for pedestrian bridge), and these operations can require minutes or hours, so a critical question is the stability in time both of the radar equipment and environmental conditions. A temperature change, for example, between morning and evening, can give a sensible perturbation of (a) the target (bridge); (b) the air between radar and target; (c) the electronic equipment. Only the latter can be compensated with a suitable calibration procedure. Another possible source of perturbation is the movement of vehicles or other metallic objects in proximity of the view cone of the radar. This can produce phase rotation detectable as false displacements. Pieraccini et al. [5] reported a case study where the radar is used in only-range modality for testing a railway bridge before the entry into service. The case in [4] is relative to the first application of radar interferometry (by imaging both in range and cross-range) aimed to carry out a static test of a pedestrian bridge. 

**Figure 12:**
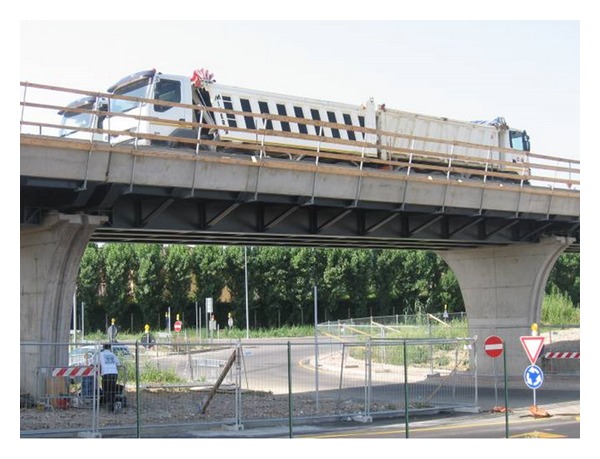
Testing of a bridge.

Static testing and dynamic testing of a bridge can be carried out even in a single measurement session as for the case shown in the picture of Figure 12. Four tucks loaded with inert materials were used as stimulus. Each weighted 44000 Kg, so the maximum applied static load was 176000 Kg.

**Figure 13:**
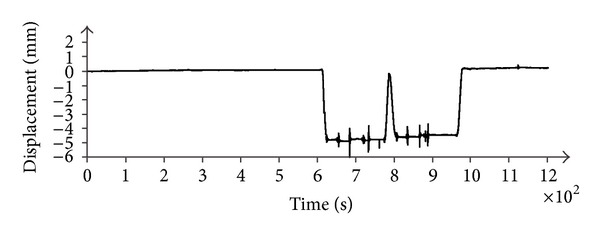
Displacement in time.

The displacement was measured by an interferometric radar that operated continuously during two load/unload cycles. The radar pointed the central part of the arcade. The measured displacement in time (Figure 13) shows that the full static load gives a displacement of about 5 mm. 

**Figure 14:**
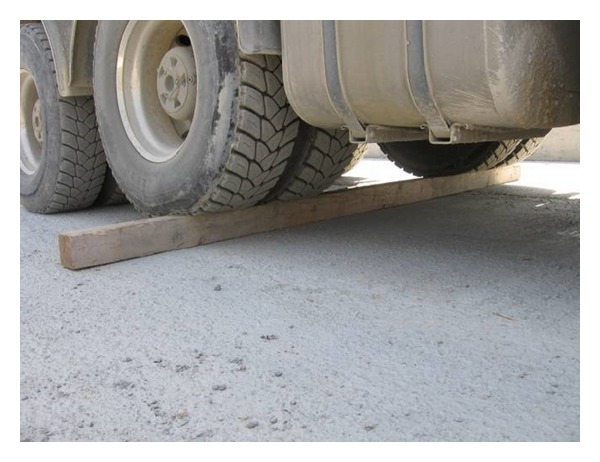
Dynamic stimulus of the bridge deck.

During the load phase, one of the trucks passed three times on a beam of wood of 15 cm side (as shown in Figure 14) soliciting the bridge with a mechanical beat.

**Figure 15:**
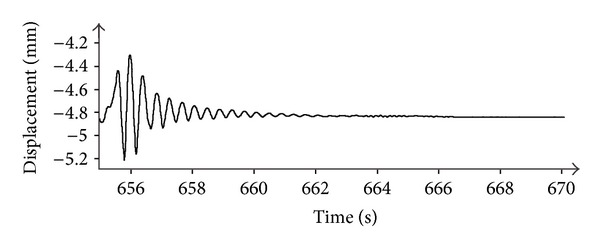
Measured pulse response in time domain of the bridge in Figure 11.

The measured response relative to a single beat is shown in Figure 15. As pulse duration is short with respect to typical movements of a bridge, the plot in Figure 15 can be considered as the pulse response in time domain of the deck, that is a function of great importance for assessing the dynamic characteristics of a bridge.

### 4.2. Dams

Dams are critical infrastructures that need continuous monitoring. In effect, one of the first applications of ground-based radar interferometry was just in a dam [25]. Many other cases are reported in literature [26–28]. The radar is able to give a displacement image projected on a horizontal (or vertical) plane. This is of valuable interest for obtaining the strain pattern of the structure. 

### 4.3. Multistorey Buildings

Static testing by radar has been assessed even in structure like multistorey buildings. As a significant example, we mention the case of a test-building already intentionally damaged. The radar has been able to detect the inelastic behaviour of the structure [29]. 

## 5. Summary and Discussion

Table 1 rearranges the references of this paper by dividing them by applications. Although the bibliographic research is, of course, not exhaustive, it clearly highlights the greatest number of works that has been published on the dynamic testing of bridges. In the field of static testing, the dams are the structures more often equipped with interferometric radars. 

As resumed even in Table 1, the application fields of ground-based radar interferometry are varied and numerous. Nevertheless, the practitioner should be aware of some limits and warnings. First of all, when the radar is used in only-range modality, it should be always taken in mind that the radiation lobe of a radar antenna is not a laser beam: single pixel in the radar plot could not correspond to a physical point in the structure, so it could be affected by static clutter and dynamic clutter due to disturbing targets at the same distance. A careful setup of the measurement geometry is essential for a successful use of this technique. Furthermore, a frequent cause of misunderstanding of the measurement data is due the fact that even the radar with its tripod is a mechanical structure with its movement. It could be even greater of the displacement to be detected, if the ground or the floor where the radar is installed is not stable. In these cases, a possible solution is to integrate a seismic accelerometer in the radar head [16] with the aim to remove its own movement in data processing.

The application field of interferometric radar could be even larger if it could be able to provide bidimentional images of the displacement pattern in dynamic conditions, that is, if it could be able to provide bidimentional images with a frame rate of a few of milliseconds. At the state of art, this is not possible, but a step in the right way seems to be done by Tarchi et al. [38]. They designed a coherent radar based on multiple inputs multiple outputs (MIMO) technology. Finally, a critical issue for a wide popularity of this technique, as well as of any advanced equipment, is the cost. Currently interferometric radars, also in the simplest configuration, are relatively expensive for the market of the structural testing. For this reason, a downgraded version of the device, without range discrimination capability has been proposed [12] and successfully tested in comparison with a standard interferometric radar. This solution is potentially very low in cost. 

## 6. Conclusion 

Ground-based radar interferometry is a measurement technique recently proposed for static testing and dynamic testing of infrastructures. It has the unique advantage to provide global information on the structure under test, with the drawback that it is often not easy to localize with precision in the structure the measured displacement as all the points in the same resolution cell can contribute to it.

The technique has been demonstrated effective in bridges, culverts, dams, towers, chimneys, pylons, antenna masts, lighting towers, streetlight, and even multistorey buildings. For all these application the radar is a valuable tool for contributing to assess the health of the structure; nevertheless it should be clearly stated that the measurements of displacements and natural frequencies, as well as the modal shapes, never give directly structural information. The health evaluation of a civil structure always depends essentially on the reliability of the specific (static or dynamic) model. The instrumental data are only possible inputs, as well as the survey and the materials tests.

## Figures and Tables

**Table 1:** 

	Static testing	Dynamic testing
Bridges	[4, 5]	[3, 5–12]
Masonry towers		[13–16]
Wind turbines		[17–19]
Chimneys		[10, 20–22]
Antenna masts		[20, 23]
Lighting towers		[11]
Culverts		[24]
Dams	[25–28]	
Buildings	[29]	[30, 31]
